# Choline Intake and Its Food Sources in the Diet of Romanian Kindergarten Children

**DOI:** 10.3390/nu9080896

**Published:** 2017-08-18

**Authors:** Cristian Reinhard Prelicz, Lucia Maria Lotrean

**Affiliations:** Department of Community Medicine, Iuliu Hatieganu University of Medicine and Pharmacy, Victor Babes 8 Street, 400012 Cluj-Napoca, Romania; cristian_reinhard@yahoo.com

**Keywords:** choline intake, children, Romania

## Abstract

The objective of this study is to assess the usual intake and food sources of choline in a group of Romanian kindergarten children. A cross-sectional study was performed among 71 children aged 4–6 years from four kindergartens from Cluj-Napoca, Romania. Dietary intake data were collected by means of three-day food records. The mean (SD) daily intake of choline was 215 (32) mg/day, 22.5% of the participants fulfilling the adequate intake (AI) for children 4–6 years of age of 250 mg of choline per day. The main food sources were meat (mainly poultry), eggs, grains, cereals, and baked products (mainly bread), and dairy products (mainly milk). The results of the logistic regression analyses show that an appropriate consumption of choline/day was statistically significantly associated with the consumption of at least one egg per three days (OR = 7.5, *p* < 0.05), a minimum of two portions of milk or dairy products per day (500 mL milk or yoghurt, or 60 g of cheese/day) (OR = 4.4, *p* < 0.05), and at least one portion of meat/day (90 g/day) (OR = 14.4, *p* < 0.05). The results underline the need for future surveys in this field, as well as actions to encourage an appropriate diet for children, including an appropriate content of choline.

## 1. Introduction

The US Institute of Medicine has recognised choline as an important nutrient for human health since 1998 when an adequate intake (AI) was set, since sufficient evidence was not available at the time for calculating an estimated average requirement and recommended daily allowance [1,2]. In the body, choline is involved in lipid transport and metabolism, methyl group donation, and the synthesis of acetylcholine and membrane phospholipids [2,3,4,5]. Several studies have recently investigated the role of choline in neurodevelopment and cognitive function, as well as the pathogenesis of various chronic diseases, including cancer and cardiovascular disease [2,4,5,6]. Choline deficiency causes fatty liver as well as liver and muscle damage, and is probably related to neoplasia and cognitive impairment [6,7].

Choline can be created through de novo synthesis, but it is predominantly obtained from the diet, because the amount of biosynthesis that occurs in the body cannot sufficiently meet the daily requirements for humans, especially during critical periods of rapid development [5,8].

The US Institute of Medicine (IOM) (which is now the Health and Medicine Division of the National Academies of Sciences, Engineering and Medicine) set the adequate intake for total choline (AI), which depends on age and sex [8,9]:

- Children: 200 mg/day for children aged 1–3 years; 250 mg/day aged 4–8 years; and 375 mg/day aged 9–13 years;
- Adolescents aged 14–18 years: 400 mg/day (girls) and 550 mg/day (boys);
- Adults older than 19 years: 425 mg/day (women) and 550 mg/day (men); and
- Special groups: 450 mg/day for pregnant women or adolescents and 550 mg/day for lactating women or adolescents.

In 2016, the European Food Safety Authority established the following recommendations for adequate intake levels for choline [6]:

- Children: 140 mg/day for children aged 1–3 years; 170 mg/day aged 4–6 years; 250 mg/day aged 7–10 years; and 340 mg/day aged 11–14 years;
- Adolescents: 400 mg/day aged 15–17 years; and
- Special groups: 480 mg/day for pregnant women.

Choline is present in a wide variety of foods, with eggs and meat products being the food products with the richest content in choline [10]. The United States Department of Agriculture (USDA) developed—and is constantly upgrading—a database that provides researchers and consumers with data about the choline content in foods [10].

Several studies have tried to assess choline intake and its food sources among different population groups from different parts of the world [5,7,11,12,13,14,15,16]. One study assessed the choline intake of the European population considering the European Food Safety Authority European Comprehensive Food Consumption Database and the United States Department of Agriculture Nutrient Database. It included data from surveys performed between 2000 and 2012 in nine European countries: Finland, Germany, France, Italy, Latvia, Ireland, Sweden, The Netherlands, and the UK. Average choline intake ranges were 151–210 mg/day among toddlers (1 to ≤3 years old), 177–304 mg/day among other children (3 to ≤10 years old), 244–373 mg/day among adolescents (10 to ≤18 years old), 291–468 mg/day among adults (18 to ≤65 years old), 284–450 mg/day among elderly people (65 to ≤75 years old), and 269–444 mg/day among very elderly people (≥75 years old) [5].

In several countries from Eastern Europe (including Romania), there is insufficient information with regard to nutrient consumption of different population groups, and there is no information available with regard to choline intake. 

Hence, this study focuses on the usual intake of choline among Romanian children aged 4–6 years. It has three objectives: The first one is to assess choline intake among Romanian children. The second one is to identify commonly-consumed foods that contribute significantly to the usual intake. Finally, factors which influence choline intake will be evaluated.

## 2. Methods

### 2.1. Study Sample and Procedure

A cross-sectional study was performed in four kindergartens from Cluj-Napoca (a large city from Northwest Romania) in April–May (in two kindergartens) and September–October 2011 (in another two kindergartens). Approval for the study was received from the review committee of each kindergarten, the standard procedure at that time in Romania. 

Parents of the children older than four years from the four kindergartens were informed that the kindergarten would be performing a study assessing the food intake of their children consisting of two parts: (a) recording information by a member of the research team regarding food intake by children on the property of the kindergarten; and (b) the second one comprised of recording the food intake of children at home by one of the parents. One-hundred and fifty parents offered informed consent for participation. Later on, 71 parents also offered detailed reliable information regarding food intake of their children (70 parents did not fill in the food records, while the others offered general information, without including detailed information regarding the type and quantities of consumed food). Hence, the final study sample consisted of 71 healthy children aged 4–6 years (34 boys and 37 girls).

### 2.2. Instruments for Data Collection

Dietary intake of choline was quantified using three-day food records completed prospectively over two weekdays and one weekend day. During the weekdays, the food consumed in kindergarten by each child was recorded by a member of the research team with the help of the personnel from the kindergarten, while the food consumed at home was recorded by parents. During weekend days, the information was recorded by parents. The parents were asked to weigh and record as detailed as possible the food and beverages consumed by their children during the investigated period of time.

Information regarding the choline content of foods was sourced from the US Department of Agriculture Database for Choline Content of Common Foods [10]. This database provides information on the total choline content of US foodstuffs, calculated as the sum of five choline-contributing metabolites: free choline, glycerophosphocholine, phosphocholine, phosphatidylcholine, and sphingomyelin [10]. 

We identified 293 distinct food items consumed by children and, as with other studies, their match with food items from the USDA Database for Choline Content of Common Foods was made based on (1) the same or similar food names; (2) similar nutrient profiles; (3) comparable physical properties; and (4) similar ingredients and preparation. Similar with other studies, commercial food products and foods unique to Romania that were not found in the USDA databases were assigned choline values from substitute foods with similar ingredients [4,5,7,16].

Energy intakes were estimated from food and beverages consumed using the Romanian food composition data and the information of the USDA National Nutrient Database, because the Romanian composition food table has limited information (it has not been updated in the last 27 years despite an increase of food items available on the market, and generally offers information regarding raw food) [17,18]. For the majority of food items, there information used was from the USDA National Nutrient Database, except some traditional food products, such as some types of cheese or meat and fat products (e.g., salami, sausages).

### 2.3. Data Analyses

First, daily intake of choline was calculated for each participant. Choline intake per day was calculated for each participant in the following way: (a) first, the choline content of the food consumed by children during the three days was calculated by multiplying, for each food item, the consumed quantity by the corresponding choline content; (b) the calculated intakes throughout the two weekdays were summed up, multiplied by five, and divided by two (A); (c) the calculated intake throughout the weekend day was multiplied by two (B); (d) The results obtained previously were summed up and divided by seven, according to the following formula: (A + B)/7. The percentage of children who fulfilled the recommended daily intake of 250 mg/day was calculated.

Second, the mean as well as the 5th, 50th, and 95th percentiles of choline intake were derived for the whole sample, as well as separately for boys and girls. An independent sample *t*-test was conducted in order to compare the mean intake of choline among boys and girls.

Third, the energy intake was calculated for each participant according to the following procedure: (a) energy content of the food consumed by children during the three days was calculated by multiplying, for each food item, the consumed quantity by the corresponding energy content; (b) the calculated intakes throughout the two weekdays were summed up, multiplied by five, and divided by two (A); (c) the calculated intake throughout the weekend day was multiplied by two (B); and (d) the results obtained previously were summed up and divided by seven according to the following formula: (A + B)/7.

Fourth, the mean, as well as the 5th, 50th, and 95th percentiles of energy intake were calculated for the whole sample, as well as separately for boys and girls. An independent sample *t*-test was conducted in order to compare the mean intake of energy among boys and girls.

Fifth, choline intake/kcal was calculated for each person by dividing the choline intake/day by the daily energy intake, and then the mean choline intake/kcal was calculated for the whole group.

Sixth, similar with other studies, the determination of the food categories that represented the highest percent contribution to dietary choline was calculated using the following formula: ((Total choline provided by food category)/(Total choline from all food categories)) × 100 [4,16].

Seventh, four logistic regression analyses were performed in order to assess the factors associated with the consumption of more than 250 g of choline daily. The dependent variable in all logistic regressions was choline daily intake at the individual level (0: less than 250 mg/day, 1: minimum of 250 mg/day). In the first regression analysis, the independent variable was daily energy intake. In the second and third regression analyses, the independent variables were consumption of at least one egg per three days (0: no; 1: yes), respectively, and consumption of at least one portion of meat (around 90 g of red meat or poultry or fish or meat products such as sausages) per day (0: no; 1: yes). Eggs and meat products are the richest dietary sources of choline, with one egg containing approximately 130 mg choline, while the content of meat products varies between 41 and 110 mg/100 g, while the content is even higher for organs (e.g., liver) [10]. Moreover, several organizations recommend the consumption of one portion of meat products or substitutes (around 90 g of meat per day) by young children as an important part of a healthy diet [19,20]. In the fourth regression analysis, the independent variable was the consumption of at least two portions of milk or dairy products per day (one portion being represented by 250 mL of milk or yoghurt or 30 g of cheese) (0: no; 1: yes). Although milk and dairy products are not rich sources of choline (16 mg of total choline per 100 g of milk, while the content of choline per 100 mg cheese varies between 14 and 36 mg depending on the type of cheese), their frequent consumption might contribute to the daily choline intake; several organizations recommend the consumption of two portions of dairy products daily as part of healthy nutrition [19,20].

Data were analysed using SPSS-20 statistics program (IBM Corp., Armonk, NY, USA). Statistical significance is reported at *p* < 0.05.

## 3. Results

The results of the study show that 22.5% of the children had a daily intake of at least 250 mg of choline, while 90.1% had at least 170 mg choline/day in their diet.

The mean daily intake was 215 mg/day (SD = 32), without noticing statistically significant differences between boys and girls (see Table 1). 

The mean energy intake was 1581 kcal/day (SD = 229), without noticing statistically significant differences between boys and girls.

On average, children had an intake of 0.14 mg choline/kcal (SD = 0.03).

Table 2 shows that the main food sources of choline were meat and meat products (the most important contributor being poultry, while fish has a very small contribution), eggs, as well as grains, cereals, baked products (bread being the main contributor), and dairy products (mainly milk). These four food groups contributed to 75% of the choline intake. Fruits, vegetables, and potatoes also contributed to 11% of the choline intake, while sweets (cakes, biscuits, chocolate, and confectionaries) contributed 7%.

Boys and girls had similar percentages for the main food sources of choline (see Table 2).

Around half of the children (46.5%) declared eating at least one egg per three days, 35.2% consumed a minimum of two portions of milk or dairy products per day, and 60.5% consumed at least one portion of meat and meat products per day.

The results of the logistic regression analyses show that an appropriate consumption of choline/day was statistically significantly associated with consumption of at least one egg per three days (OR = 7.5, *p* < 0.05), a minimum of two portions of milk or dairy products per day (OR = 4.4, *p* < 0.05), and at least one portion of meat per day (OR = 14.4, *p* < 0.05). The daily energy intake was not statistically significantly associated with choline intake.

## 4. Discussion

This study offers information regarding choline intake and its food sources among Romanian kindergarten children aged 4–6 years. The results show that average choline intake was 215 mg/day. European surveys performed in nine European countries showed that the average choline intake varied between 177 and 304 mg/day among three to ≤10 year-old children [5].

Only 22.5% of the children included in our study fulfil the recommended adequate intake by the US Institute of Medicine of 250 mg/day. 

Eggs and meat products are the richest dietary sources of choline [10]. In our population group, eggs and meat products (mainly poultry) were the source of more than 40% of the choline intake of the children. Moreover, children who ate at least one egg per three days and at least one portion of meat/day, respectively, had a significantly higher chance of fulfilling the recommendations of choline intake. Studies from other countries also underline this [16].

Although milk and dairy products are not a rich source of choline compared with eggs, they are consumed in significant quantities by many children and they therefore represented a major food source of choline [5,16]. Moreover, similar with other studies, the results show that children who consumed at least two portions of milk or dairy products/day had a higher chance of fulfilling the recommendations [16].

Bread, pasta, cereals, and grains have a moderate content in choline, but because of their frequent consumption, they contributed 16% of the total choline intake. Studies from other European countries showed that these products can contribute 6.4–21.6% of the total choline intake [5].

Fruits, vegetables, and potatoes, but also several sweets, have low or moderate choline content, but because of the consumed quantities they succeeded in contributing to 19% of the choline intake.

This study is subject to some limitations. First, the sample is limited to kindergarten children from several kindergartens of a large city in Romania, and has no national representativeness, while the refuse rate of participation of parents was high, possibly leading to a selection of children coming from families who are paying more attention to healthy nutrition. Second, the information was obtained through a three-day food record, but for the food consumed at home it relies on the capacity and motivation of parents to make detailed and correct records. This might lead to either underestimated or overestimated food consumption for some children. Third, similar with other studies, because there is no food composition data for choline available from Europe, data from the US were used [5].

## 5. Conclusions

To the best of our knowledge, this is the first study investigating choline intake among a Romanian population, and one of the few studies from Europe investigating this issue among 4–6 year old children. The results underline the need for future surveys in this field as well as the necessity to educate, motivate, and help kindergarten professionals in preparing food, and assisting parents with regard to ways of assuring appropriate nutrition for children, including a good content of choline. As recommended by several organizations, appropriate consumption of meat products, dairy products, and eggs increases the chance of appropriate choline intake among kindergarten children [19,20].

## Figures and Tables

**Table 1:** Total choline and energy intake.

	Min.	Max.	Mean (SD)	95% Confidence Interval	Percentiles 5th	Percentiles 50th	Percentiles 95th
Choline mg/day							
Total (*N* = 71)	142	288	215 (32)	207–222	159	215	265
Girls (*N* = 37)	142	288	215 (37)	203–227	153	218	283
Boys (*N* = 34)	143	258	214 (26)	204–222	167	210	258
Energy (kcal/day)							
Total (*N* = 71)	1246	2377	1581 (229)	1530–1638	1297	1539	2026
Girls (*N* = 37)	1246	2063	1578 (224)	1509–1648	1308	1539	2006
Boys (*N* = 34)	1270	2377	1585 (237)	1512–1666	1287	1543	2221

**Table 2:** Main food sources of total choline.

Food Product	Percentage of the Total Choline		
	Total	Boys	Girls
Dairy products	15.5	15.2	15.8
Milk	7.5	7.3	7.7
Yoghurt	2.5	2.2	2.8
Cheese	4	4.2	3.8
Sour cream and butter	1.5	1.7	1.3
Meat/Poultry/Fish	23.3	24.3	22.3
Red meat	7.6	8.3	6.9
Poultry	9.4	9.2	9.6
Fish	0.9	1	0.8
Sausages/salami/ham/organs (pate)	5.4	5.8	5
Eggs and egg products	20.6	21	20.2
Grains, cereals, baked products	16.2	15.6	16.8
Bread	6.5	6.9	6.1
Pasta	3.1	2.9	3.3
Cereals	4.1	3.7	4.5
Grains, flour, rice	2.5	2.1	2.9
Fruits/vegetables/potatoes	11.4	10.2	12.6
Fruits (fresh, canned, juices)	3.5	3.2	3.8
Vegetables (fresh, cooked, juices)	5.6	4.6	6.6
Potatoes (fried, boiled)	2.3	2.4	2.2
Sweets	7.5	7.8	7.2
Cakes, biscuits	3.4	3.8	3
Chocolate, ice-cream, pudding	2.6	2.3	2.9
Sugar, jam, and confectionery	1.5	1.7	1.3
